# Historical and Contemporary Evidence Confirms a Higrevirus as the Causal Agent of Citrus Zonate Chlorosis in Brazil

**DOI:** 10.3390/v17111428

**Published:** 2025-10-28

**Authors:** Laura R. Pereira, Mariane C. Rodrigues, Camila Chabi-Jesus, Pedro L. Ramos-González, Cristiane J. Barbosa, Magno G. Santos, Helcio Costa, Luana C. Maro, Aline D. Tassi, Elliot W. Kitajima, Ricardo Harakava, Juliana Freitas-Astúa

**Affiliations:** 1 ULR em Biologia Molecular Aplicada, Instituto Biológico, Av. Cons. Rodrigues Alves, 1252, São Paulo 04014-002, SP, Brazil; macrodrigues12@gmail.com (M.C.R.); plrg1970@gmail.com (P.L.R.-G.); ricardo.harakava@sp.gov.br (R.H.); juliana.astua@embrapa.br (J.F.-A.); 2 Plant Virus Vector Interactions Laboratory, Entomology & Plant Pathology Department, North Carolina State University, Raleigh, NC 27695-7103, USA; millachabi@yahoo.com.br; 3 Embrapa Mandioca e Fruticultura, Cruz das Almas 44380-000, BA, Brazil; cristiane.barbosa@embrapa.br (C.J.B.); magno.santos@embrapa.br (M.G.S.); 4 Instituto Capixaba de Pesquisa, Assistência Técnica e Extensão Rural (INCAPER), Rua Afonso Sarlo, 160, Vitória 29052-010, ES, Brazil; helciocosta@incaper.es.gov.br; 5 Empresa de Pesquisa Agropecuária e Extensão Rural de Santa Catarina (EPAGRI), Estação Experimental da Epagri, Itajaí 88318-112, SC, Brazil; luanamaro@epagri.sc.gov.br; 6 Tropical Fruit Entomology Laboratory, Tropical Research and Education Center, University of Florida, Homestead, FL 33031-3314, USA; alinetassi@gmail.com; 7 Laboratório de Microscopia Eletrônica Aplicada à Pesquisa Agropecuária, Escola Superior de Agricultura Luiz de Queiroz (ESALQ), Universidade de São Paulo, Piracicaba 13418-900, SP, Brazil; ewkitaji@usp.br

**Keywords:** kitavirids, herbarium specimens, *Brevipalpus* mites

## Abstract

Citrus leprosis (CL) and citrus zonate chlorosis (ZC) were first described in Brazil in the 1930s. Both diseases, which caused non-systemic lesions primarily characterized by chlorotic and/or necrotic spots, were associated with the presence of *Brevipalpus* mites. While CL has since been well characterized as being caused by viruses of the genera *Cilevirus* (family *Kitaviridae*) and *Dichorhavirus* (family *Rhabdoviridae*) and transmitted by several species of *Brevipalpus* mites, the causal agent of ZC remained unknown. In this study, we analyzed *Citrus* spp. samples exhibiting typical ZC symptoms using high-throughput sequencing (HTS) to determine the etiology of ZC. We examined historical herbarium specimens collected between 1933 and 1965 alongside fresh samples collected from 2016 to 2022. Our results identified the higrevirus hibiscus green spot virus 2 (HGSV2, *Higrevirus waimanalo*) as the causal agent of ZC. In addition, we report for the first time the presence of a higrevirus in continental America, expand the diversity of known kitaviruses infecting citrus in Brazil, and demonstrate the transmission of an higrevirus by *Brevipalpus yothersi* and *B. papayensis*.

## 1. Introduction

Brazil is the world’s leading producer of sweet oranges. The vast majority of these fruits are processed into juice, accounting for around 60–70% of global orange juice production [1]. In addition to sweet oranges, the country also produces over 1 million tons of mandarins, mainly destined for the domestic fresh market, ranking as the world’s 5th largest mandarin producer in 2024 [2,3]. Brazilian citrus production is recurrently affected by unfavorable weather conditions and diseases caused by bacteria, fungi, and viruses. Among viral diseases, citrus leprosis (CL) is the most significant, leading to early fruit drop of almost 3% of the total number of boxes produced at its peak in 2022 [4]. CL is caused by viruses transmitted by Brevipalpus mites, which are classified into two genera: *Cilevirus* and *Dichorhavirus* [5]. The disease was first reported in Brazil in 1931 [6], the same decade that another disease, known as citrus zonate chlorosis (ZC), was described in the country [7,8]. ZC was associated with an unknown pathogen, likely a virus, causing non-systemic chlorosis, with rare or no necrosis on citrus leaves, symptoms that could also be mistakenly associated with CL. At the time, ZC was considered the most important disease affecting Brazilian citrus production in the State of Rio de Janeiro [9]. Consequently, efforts were made to identify its causal agent and vector. In the 1960s, ZC was demonstrated to be transmitted by *Brevipalpus* mites [10,11]. Samples of citrus plants naturally infected with ZC, as well as plants used in transmission assays with *Brevipalpus* sp. between 1933 and 1969, were preserved in the herbarium of the Instituto Biológico in São Paulo, Brazil. In the following decades, however, although never eradicated, the relevance of ZC diminished as other diseases became more important to the Brazilian citrus industry, and research on the disease was discontinued.

In 2012, Volkamer lemon (*Citrus volkameriano*) and *Hibiscus arnottianus* plants exhibiting ring spots resembling those described for ZC and CL were reported in Hawaii, USA. Subsequent analyses of these plants revealed the presence of hibiscus green spot virus 2 (HGSV2, *Higrevirus waimanalo*, family *Kitaviridae*) [12]. Viruses in the genus *Higrevirus* are characterized by a tripartite (+) ssRNA genome, in contrast to the two- and four-segmented genomes of cileviruses and blunerviruses, respectively [13]. The RNA1 segment of HGSV2 is ~8 kb long and contains two ORFs coding for the replication-associated RdRp and an orphan protein of 11 kDa with two predicted transmembrane domains [12]. The *RdRp* gene includes conserved domains for methyltransferase, cysteine protease, helicase and RdRp-2. The RNA2 is ~3 kb long, tetracistronic, and encodes three putative proteins. ORFs *p39* and *p9*, located at the 5′-end of the genomic segment, are also termed *BMB1* and *BMB2* and are necessary and sufficient to mediate cell-to-cell viral movement [14]. RNA3 of the first isolate of HGSV2 described (HGSV2_WAI 1-1) is ~3 kb long and has three ORFs encoding proteins of 33, 29 and 23 kDa. The protein encoded by *p33* belongs to the SP24 family (PF16504), whereas the other two proteins share very low identity values with known proteins [12]. Two additional isolates from Hawaii were later characterized by HTS and the composition of their RNA3 differed from that of HGSV2_WAI 1-1. In these new isolates, RNA3 contains two putative overlapping ORFs instead of the three previously reported [15]. It was also determined that HGSV2 also naturally infects *C. reticulata*, *C. sinensis*, *H. tiliaceus*, and the experimental plants *Phaseolus vulgaris*, *Nicotiana tabacum* and *N. benthamiana* [15]. To date, HGSV2 had only been detected in three islands of Hawaii and has been shown to be transmitted by *Brevipalpus azores* [12,15].

In this study, we determined the pathogen associated with ZC disease through HTS analysis of symptomatic citrus samples collected between 2016 and 2022 from the Active Germplasm Collection of Embrapa Cassava and Fruits, Cruz das Almas, Bahia, Brazil. Additionally, virus genomic sequences were recovered from the sequencing of small RNA fraction extracted from leaf samples preserved in the herbarium of the Instituto Biológico, São Paulo, Brazil. One of these samples was collected from symptomatic plants infested with viruliferous Brevipalpus mites during a transmission experimental assay carried out in 1965 [11].

## 2. Materials and Methods

### 2.1. Plant Material and Transmission Electron Microscopy

A total of 21 *Citrus* spp. samples originated from four Brazilian states were analyzed in this study. The observed symptoms were, mostly chlorotic ring spots, often associated with the central vein. Additionally, isolated circular chlorotic lesions were found, as well as chlorotic ring spots in *C. reticulata* fruits collected in Domingos Martins, ES. Besides the fresh samples collected between 2016 and 2022, we also examined herbarium samples from the Instituto Biológico, dating from 1933 to 1965 (Table 1).

An exemplary sample of *C. aurantium* was analyzed through transmission electron microscopy (TEM). Thin sections of symptomatic tissue were cut with razor blades and fixed in Karnovsky’s solution (2.5% glutaraldehyde and 2% paraformaldehyde in 0.05 M cacodylate buffer pH 7.2) [16]. The samples were prepared and analyzed with a Jeol JEM 1011 microscope (JEOL, Tokyo, Japan), as previously described [17].

### 2.2. RNA Extraction and HTS Preparation

The preparation and analysis of fresh samples collected after 2016 (Table 1) followed previously established protocols [18]. In brief, approximately 500 ng of purified RNA extracts were sent to the Animal Biotech Laboratory at the Escola Superior de Agricultura Luiz de Queiroz, University of São Paulo (Piracicaba, SP, Brazil) for sequencing using the HiSeq 2500 platform (Illumina, San Diego, CA, USA). Poly(A)-tail enrichment and cDNA library construction were performed with the Illumina TruSeq Stranded mRNA Library Prep Kit (Illumina, San Diego, CA, USA). Sequencing was conducted on the HiSeq 2500 platform, generating 150 bp paired-end reads with the HiSeq SBS v4 High Output Kit (Illumina, San Diego, CA, USA).

To investigate herbarium specimens with zonate chlorosis collected between 1933 and 1965 (Table 1), the small RNA (sRNA) fraction was subjected to HTS using the Ion Torrent platform (Thermo Fisher Scientific, Waltham, MA, USA), as previously applied to herbarium specimens and Brevipalpus Transmitted Viruses (BTVs) [18]. sRNAs extraction and purification were performed with the PureLink™ miRNA Isolation Kit (Thermo Fisher Scientific) according to the manufacturer’s recommendations. RNA integrity was assessed with a Bioanalyzer. sRNA enrichment and library construction were carried out with the Ion Total RNA-Seq kit v2 for Small RNA libraries (Thermo Fisher Scientific). Libraries were loaded onto chips using the Ion 540^®^ Kit-Chef and sequenced on the Ion GeneStudio™ S5 System. For all samples analyzed by HTS, as well as those not subjected to HTS, approximately 500 ng of total RNA was utilized for cDNA synthesis with the GoScript^®^ Reverse Transcriptase kit (Promega, Madison, WI, USA) and random primers. For validation and viral detection, PCR assays were performed with 1 µL of synthetized cDNA and GoTaq^®^ Green Master Mix (Promega).

### 2.3. Bioinformatic Analysis

Seven HTS libraries were generated in this work. Data analysis followed established workflow [18]. Read quality was assessed with FastQC [19], and adapter sequences were removed with Trimmonatic for the data derived from Illumina sequencing [20]. De novo genome assembly was performed with SPAdes (via Geneious Prime v.2021.0.1; Geneious, Auckland, New Zealand), applying different k-mer sizes for sRNA and mRNA libraries, as previously described [18]. The resulting contigs were identified with BLASTx/BLASTn implemented in Geneious Prime v.2021.0.1, using RefSeq (NCBI) [21] and additional unpublished sequences from the Laboratory of Applied Molecular Biology at Instituto Biológico, SP, Brazil. When necessary, the obtained reads were mapped to the reference genome of isolate HGSV2 WAI 1-1 [12] using Bowtie2 implemented in the Galaxy platform [22,23]. Viral ORFs were predicted with ORFinder (NCBI), translated, and analyzed for the presence of signal peptides, conserved domains and transmembrane regions. The presence of signal peptides was predicted with SignalP v.6.0 [24], conserved domains were identified using MOTIF Search (https://www.genome.jp/tools/motif/, accessed on 16 September 2025); transmembrane helices were predicted with DeepTMHMM 1.0 [25] and Deeploc v.15 [26], as previously described [27].

For molecular validation of HTS results, primers were designed based on isolate DMs_01 to generate overlapping amplicons spanning the complete HGSV2 genome (Supplementary Table S3). Using these primers, amplicons covering RNA1, RNA2 and RNA3 were obtained from isolate CdA_10 and sequenced by Sanger sequencing. Sequences were aligned with CLUSTAL W implemented at Vector NTI (Thermo) v. Advanced v.10.

### 2.4. Phylogenetic Analysis

For phylogenetic analysis, amino acid sequences of the RdRp protein from all accepted viruses of the family Kitaviridae were aligned using MAFFT v. 7 [28]. A maximum-likelihood tree was inferred with IQ-TREE [29] under the LG+F+I+G4 model [30] with 1000 bootstrap replicates [31]. The tree was visualized and edited in the Interactive Tree of Life (iTOL) [32].

### 2.5. Morphological Identification of Brevipalpus Mites and Transmission Assays

In samples collected in 2021 from Cruz das Almas, BA (isolate CdA_12), the presence of *Brevipalpus* mites was visually verified using an optical magnifying glass. In addition to live mites, carcasses of eight specimens and eggs were identified. Eggs were maintained on *C. sinensis* plants until hatching; adult mites and carcasses were fixed in 70% ethanol, mounted, and examined for morphoanatomical analysis with a Zeiss AxioImager D2 microscope (Carl Zeiss AG, Jena, Germany), as previously reported [17]. For scanning electron microscopy, mites were dehydrated in ethanol series, and sample preparation and visualization followed established protocols [33]. Taxonomic identification was based on morphological criteria described for the taxa assignation within the genus *Brevipalpus* [34].

To assess transmission of the virus associated with zonate chlorosis, *Brevipalpus* mites were used in assays with *Arabidopsis thaliana* Col-0 plants. The experimental plant is considered a host for both *Brevipalpus* mites and for the viruses they transmit [35]. Three experimental conditions were established: Experiment I—*Brevipalpus* sp. mites collected directly from symptomatic *C. reticulata* leaves (Itj_01) were transferred to 14 leaves of four arabidopsis plants under an optical microscope with a single fine brush, with 5 mites per leaf; Experiment II—approximately 150 individuals of an isoline population of *B. yothersi* maintained on *Canavalia ensiformis* were transferred to symptomatic leaves of *C. reticulata* (isolate CdA_15) for a 4-day acquisition access period (AAP) and then moved to 26 leaves of 23 arabidopsis plants (5 mites per leaf); Experiment III—around 300 individuals from a *B. papayensis* population maintained on *Coffea* sp. were transferred to symptomatic *C. reticulata* leaves (isolate CdA_15) for a 4-day AAP and subsequently transferred to 66 leaves of 10 arabidopsis plants (5 mites per leaf).

In all experiments, *A. thaliana* Col 0 plants of approximately three weeks old were maintained in a growth chamber (Adaptis AR A1000; Conviron, Winnipeg, MB, Canada) at 23 ± 1 °C with a 12 h photoperiod. At 10 to 12 days post-infestation, infested leaves were collected, and total RNA was extracted as described above. Prior to RNA extraction, mites and eggs were carefully removed from the symptomatic leaves to avoid contamination. Viral presence in the collected leaves was assessed by RT-PCR using RdRp-specific primers [12].

## 3. Results

### 3.1. Symptom Characterization and Microscopy Suggest a Viral Agent Is Associated with Zonate Chlorosis

**Figure 1:**
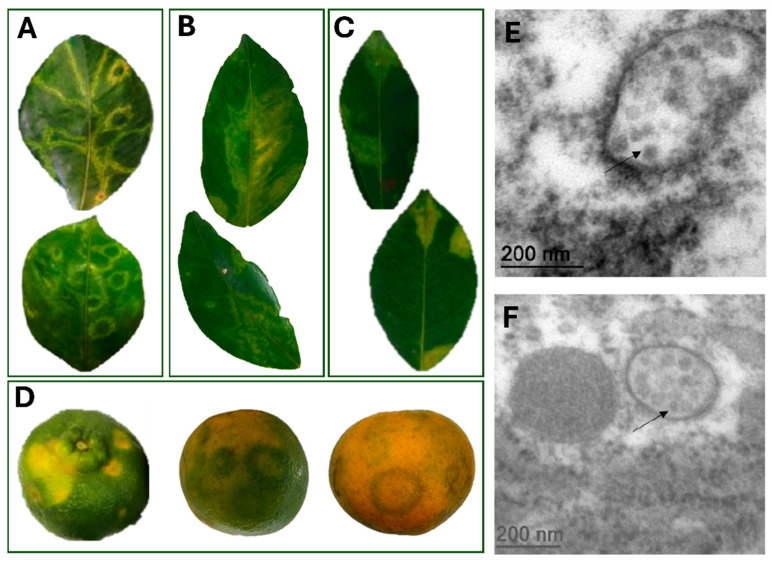
Symptoms of zonate chlorosis on citrus samples and transmission electron microscopy analysis. (**A**–**C**). Leaves of *Citrus* sp. samples showing diverse symptom patterns associated with zonate chlorosis. (**A**). Chlorotic ringspots with bright green and yellow-green zones throughout the leaf of *C. sinensis* from the Active Germplasm Bank of Embrapa Cassava and Fruits, Cruz das Almas, BA, 2016 (CdA_01). (**B**). Chlorotic blotch running along both sides of the midvein, often referred to as “oak-leaf pattern” observed in *C. sinensis* samples of isolate CdA_01. (**C**). Circular chlorotic spots with semi-regular edges, randomly distributed on the leaf of *C. reticulata* from Dominos Martins, ES, 2017 (DMs_01). (**D**). Fruits of *C. reticulata* from Itajaí, SC, 2022 (isolate Itj_02) exhibiting chlorotic ringspots without necrotic tissue. In green fruits, yellow blotches are visible, whereas in maturing fruits symptoms appear as dark green ringspots with thick bands. In mature mandarin fruits, light green ringspots with thin bands are observed. (**E**,**F**). Transmission electron micrographs of ultrathin sections of symptomatic leaves of *C. aurantium* from 2021. (**C**): Putative virions are seen as aggregates in the cytoplasm (indicated by arrows).

All fresh *Citrus* spp. samples analyzed through HTS were received from the States of Bahia and Espírito Santo and showed typical zonate chlorosis (ZC) symptoms. These samples exhibited chlorotic ring spots, circles and/or irregular shapes, with little to no necrotic tissue (Figure 1). In one symptomatic pattern, chlorotic ring spots with light green, dark green and yellowish tones were present across the leaf, representing different inoculation foci by *Brevipalpus* mites (Figure 1A). Another distinct pattern was characterized by a chlorotic blotch running along both sides of the midvein, forming what is known as an “oak-leaf pattern” (Figure 1B). Additionally, circular chlorotic spots with semi-regular edges were also found randomly distributed on the leaf (Figure 1C). In general, fruit samples of *C. reticulata* showed symptoms of chlorotic ring spots without necrotic tissue (Figure 1D). At least two of the different symptom patterns were present on the same plant, potentially indicating distinct stages of disease development. Tissues from isolated lesions of different leaves of *C. aurantium* samples were analyzed through TEM, and viral-like particles of around 50 nm were observed (Figure 1E) in the sour orange samples.

### 3.2. HTS Reveals HGSV2 as the Causal Agent of Zonate Chlorosis in Fresh and Herbarium Specimens

To identify the causal agent associated with zonate chlorosis, HTS was performed on both fresh samples (2016–2017) and historical herbarium samples collected between 1933 and 1969 (Table 1). Details on the quality of the raw sequencing data obtained of all analyzed isolates are provided in Supplementary Table S1. Contigs with significant similarity to hibiscus green spot virus 2 (HGSV2, *Higrevirus waimanalo*) were identified through BlastX analysis (*E*-value ≈ 0) and isolates were designated based on their city of origin and chronological order of identification.

**Figure 2:**
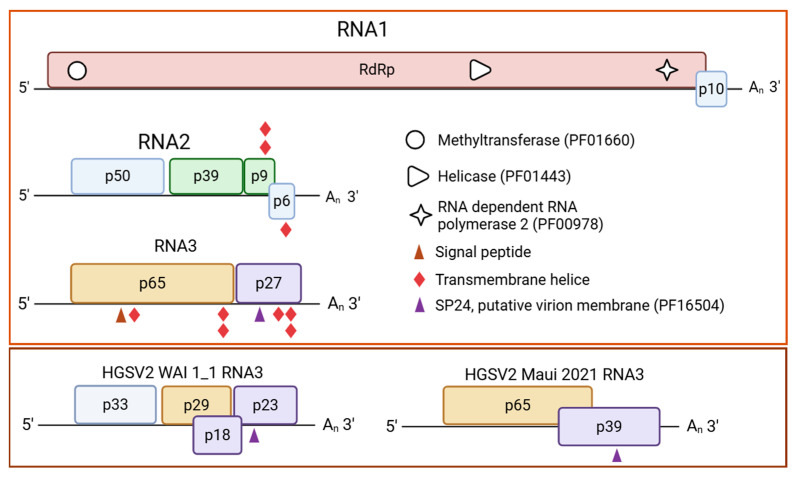
Genome representation of Brazilian isolate DMs_01 of hibiscus green spot virus 2 (HGSV2, *Higrevirus waimanalo*), the causal agent of zonate chlorosis. Boxes indicate open reading frames (ORFs), and fill colors represent conserved structural or functional features of the encoded protein. Light brown: RNA dependent RNA polymerase gene (RdRp); light green: genes coding for proteins of the binary movement block (p39/BMB1 and p9/BMB2); light orange: gene coding for the putative glycoprotein (p65); light purple: gene with a domain of the negevirus SP24-like protein (p23); light blue: ORFs with unpredictable functions. GenBank accession number of DMs_01 RNA1: OR161045; RNA2: OR161046; RNA3: OR161047. In the lower dark brown box, the RNA3 molecules of isolates WAI 1-1 (NC016142) and Maui 2021 (OQ689790) are shown to illustrate the difference in RNA3 organization compared with the Brazilian isolate DMs_01.

From the contemporary DMs_01 isolate, three contigs corresponding to the complete genomic segments of HGSV2 were recovered. RNA1, RNA2 and RNA3 were 8324 nt, 32,210 nt and 3184 nt in length, respectively, representing 100% coverage of the HGSV2 WAI 1-1 reference genome [12]. The DMs_01 isolate shares 94% nucleotide sequence identity with WAI 1-1 (Table 2) and was established as the Brazilian reference genome. It exhibits the canonical higrevirus genomic organization: two open-reading frames (ORFs) in RNA1 and four ORFs in RNA2 (Figure 2). The genomic organization of RNA3 in the DMs_01 isolate differed from that of WAI 1-1, featuring two ORFs (p65 and p27), compared to the four ORFs in the Hawaiian isolate (Figure 2). The main difference between DMs_01 and the Maui (2021) isolate was the length of the second ORF in RNA3. The recent Hawaiian isolate contains an ORF that codes for a 39 kDa protein, whereas DMs_01 contains a smaller ORF encoding a 27 kDa protein (Figure 2).

To further investigate the genetic diversity of HGSV2 in Brazil, two additional samples from the coastal states of Bahia and Espírito Santo were sequenced. In the *C. reticulata* sample (DMs_02), complete genomic segments were assembled (RNA1: 8375 nt; RNA2: 315 nt; RNA3: 3188 nt; coverage > 98%). From the *C. sinensis* sample (CdA_01), five viral contigs representing a partial genome were obtained (coverage > 93%) (Supplementary Table S2). HTS results were validated by amplifying the viral genome with primers designed from the HTS-derived sequences (Supplementary Table S3). All amplicons were sequenced, yielding a complete genome sharing 90% nucleotide identity with DMs_01.

To study the historical presence of the virus in Brazil, HTS of small RNAs (sRNAs) was conducted on herbarium specimens from the Instituto Biológico. Four specimens of *Citrus* sp., *C. reticulata*, and *C. limonia*, collected between 1937 and 1965, were analyzed (Table 1).

Partial genomes were recovered from these preserved specimens (Supplementary Table S2). From isolate Utb_01, one RNA1 contig (~260 nt) and three RNA2 contigs were recovered, while no RNA3 contigs were obtained (Supplementary Table S2). From Itn_02, five RNA1 contigs, three RNA2 contigs and two RNA3 contigs were obtained. From SPa_01, one RNA1contig (237 nt) and five RNA2 contigs were recovered; RNA3 contigs were absent (Supplementary Table S2). Notably, Spa_01 originated from a *C. limonia* sample used in an early transmission study involving *B. phoenicis* s.l. [11]. From the Cmp_01 isolate, eleven RNA1 contigs, one RNA2 contig of 2858 nt and four RNA3 contigs, the largest with 1090 nt, were recovered (Supplementary Table S2). Due to the lower coverage of Ubt_01 and Spa_01, nucleotide sequence comparison was not performed for these isolates. Comparative analysis revealed that Cmp_01 and Itn_02 shared 87.6–91.1% identity for RNA1, 93.8–95.6% for RNA2, and 90.3 to 96.2% for RNA3 against DMs_01 (Table 2). The missing genomic segments in some samples may be attributed to the high level of degradation of the material.

Across all seven HTS libraries analyzed, it was not possible to identify contigs for any other viruses besides HGVS2. This is consistent with prior sequencing studies of citrus-infecting kitavirids, where only *Brevipalpus*-transmitted viruses were recovered [17,18,36]. These results conclusively demonstrate that HGSV2 has been present in Brazilian citrus samples since at least the 1930s and is the causal agent of zonate chlorosis.

### 3.3. In Silico Analysis of the Brazilian Isolate of HGSV2

Comprehensive in silico analysis of the DMs_01 isolate revealed several conserved functional domains and features characteristic of higreviruses. Within the RdRp protein, a predicted nuclear localization signal (NLS) was identified at amino acids 1392–1408. Furthermore, the RdRp protein contains essential catalytic and structural motifs, including RdRp_2 (PF00978), viral methyltransferase (PF01660) and viral superfamily 1 helicase (PF01443). Additional domains identified include a viral superfamily 1 helicase (PF01443) domain within the P39 protein of the RNA2 molecule. In RNA3, analysis of the P61 protein predicted a signal peptide in the first 28 residues (*p* = 0.92) and a SP24 motif (putative virion membrane of plant and insect viruses, PF16504) was predicted for the P27 protein (Figure 2). Two transmembrane helices (TMHs) were identified in the P9 and P6 proteins of RNA2. In RNA3, three TMHs were identified in P61 (positions 13–35, 503–525, and 532–554) and three in the P23 protein (Figure 2).

### 3.4. Brazilian Isolate of HGSV2 Clusters with Hawaiian Isolate in the Genus Higrevirus

**Figure 3:**
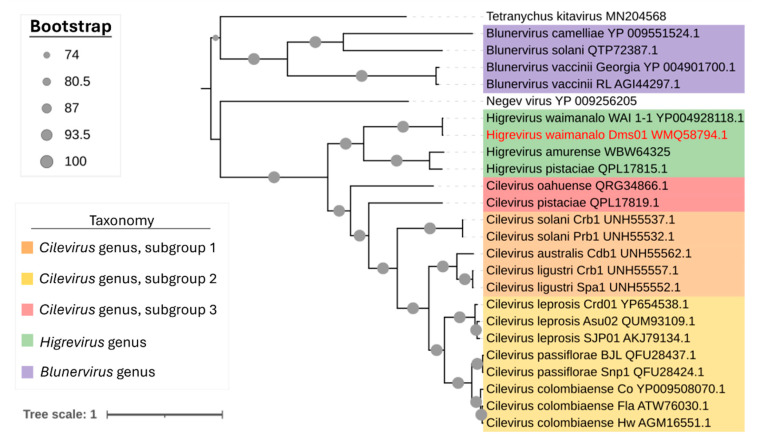
Phylogenetic relationships of the HGSV2 (*Higrevirus waimanalo*) isolate identified in Brazil with other viruses of the Kitaviridae family by a maximum likelihood tree using the RdRp proteins of all viruses. The Brazilian HGSV2 isolate is highlighted with red letters. GenBank accession numbers for each viral sequence are indicated next to the species names. Tetranychus kitavirus and Negev virus were used as outgroups.

Comparisons of RNA1 nucleotide sequences of DMs_01 against other higreviruses revealed 53 to 95% identity (Table 2). In agreement with the nucleotide identity data, the phylogenetic reconstruction based on RdRp amino acid sequences from all viruses of the family *Kitaviridae* showed DMs_01 clusters with WAI_1-1 in the branch corresponding to the genus *Higrevirus* (Figure 3).

### 3.5. HGSV2 Can Be Found in Diverse Citrus spp. Samples from Coastal Regions of Brazil, Associated with Brevipalpus Yothersi and B. papayensis

**Figure 4:**
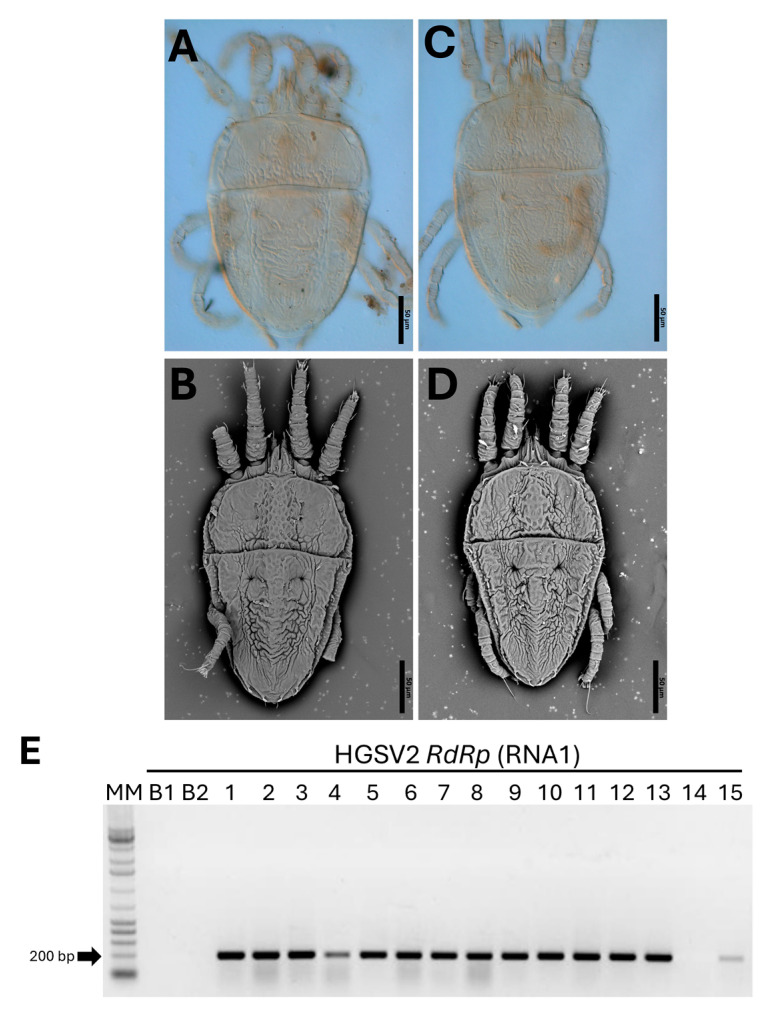
Characterization of *Brevipalpus* mites identified in citrus samples exhibiting symptoms of zonate chlorosis and detection of HGSV2 in *Citrus* spp. samples. (**A**–**D**). Micrographs of *Brevipalpus* mites collected from zonate chlorosis samples from 2016-2017. (**A**,**B**): *B. papayensis* identified by scanning electron microscopy (SEM) and light microscopy in *C. sinensis* samples from Cruz das Almas, BA, 2021 (CdA_12). (**C**,**D**). Light and SEM micrographs of *B. yothersi* identified in *C. sinensis* samples from Domingos Martins, ES, 2017 (DMs_03). (**E**). Representative detection of HGSV2 in different *Citrus* spp. samples from coastal regions of Brazil by RT-PCR using primers for the RdRp gene of the RNA1. Expected amplicon size: 198 bp. MM: Molecular marker 1 Kb Promega (Cat. G5711). B1: Blank PCR reaction. B2: Blank cDNA reaction. Sample lanes: 1—*Citrus* sp., VNI_01. 2—*C. reticulata*, DMs_01. 3—*C. reticulata*, DMs_02. 4—*C. sinensis*, DMs_03. 5—*C. sinensis*, CdA_03. 6—*C. deliciosa*, CdA_07. 7—*C. reticulata*, Itj_02. 8—*C. sinensis*, CdA_04. 9—*C. clementina*, CdA_09. 10—*C. aurantium*, CdA_10. 11—*C. sinensis*, CdA_12. 12—*C. sinensis* × *C. paradisi*, CdA_14. 13—*C. limon*, CdA_17. 14—*C. sinensis*. 15—*C. reticulata*, CdA_15.

To investigate the presence of HGSV2 in other regions of Brazil, a total of 14 *Citrus* spp. samples were tested by RT-PCR. This survey revealed a high incidence of the virus, with 20 samples testing positive (Figure 4E). The positive samples included *C. aurantium*, *C. sinensis* × *C. paradisi*, and *C. limon*, all collected from coastal regions of the States of Bahia, Espírito Santo, and Santa Catarina, corresponding to the Northeastern, Southeastern, and Southern macro-regions of Brazil, respectively. Symptomatic leaves were inspected for the presence of mite vectors. Diagnostic morphological features—including dorsal and ventral reticulation patterns, setation, palp segmentation, and leg chaetotaxy—were used for identification [34]. Based on these characters, four adults and two nymphs were identified as *B. papayensis* through light and scanning electron microscopy (Figure 4A,B), and two adults as *B. yothersi* (Figure 4C,D). Eggs and adult of *B. papayensis* mites from another *C. reticulata* fruit sample were collected for transmission assays to *Arabidopsis thaliana*. Voucher specimens were slide-mounted in Hoyer’s medium and deposited in the acarological collection of the deposited in the collection of Departamento de Entomologia, Fitopatologia e Zoologia Agrícola, Universidade de São Paulo, ESALQ, Piracicaba, São Paulo, Brazil, ensuring traceability of identifications.

### 3.6. The Causal Agent of Zonate Chlorosis Is Transmitted by Brevipalpus Yothersi and B. papayensis

**Figure 5:**
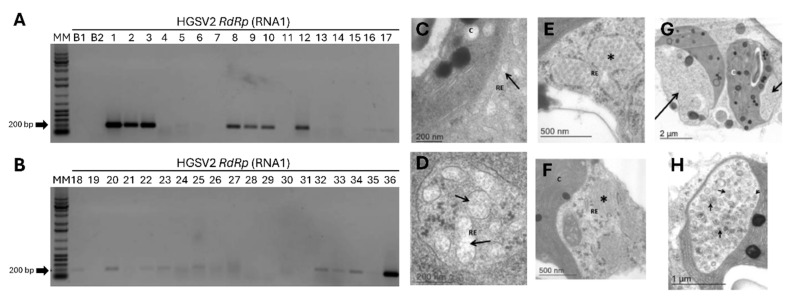
Evaluation of HGSV2 transmission by *Brevipalpus* spp. mites to *Arabidopsis thaliana*. (**A**,**B**). Representative RT-PCR results of arabidopsis leaves infested with *Brevipalpus* mites. RT-PCR assays used primers for the RdRp gene of HGSV2, with an expected amplicon of 198 bp. MM-Molecular Marker 1 Kb (Promega, Cat. G5711). B1: Blank PCR reaction. B2: Blank cDNA reaction. Lanes 1–11: Arabidopsis leaves infested with *Brevipalpus* mites taken directly from *C. reticulata* samples infected with HGSV2 (Experiment I). Lanes 12–21: Samples from Experiment II, where a laboratory-maintained population of non-viruliferous *B. papayensis* mites acquired the virus from symptomatic citrus samples and were transferred to arabidopsis leaves. Lanes 22–35: Experiment III, where a laboratory-maintained population of non-viruliferous *B. yothersi* mites acquired the virus from a symptomatic citrus sample and were transferred to arabidopsis leaves. Only some of the infested leaves used in the transmission assays are shown. Lane 36: Positive control (citrus sample infected with HGSV2). (**C**–**H**): Micrographs of ultrathin sections of arabidopsis leaves infected with HGSV2 after transmission by *Brevipalpus* spp. mites, showing unique cytopathic effects. (**C**,**D**). Arrows indicate fibrillar materials. (**E**,**F**). Asterisks (*) indicate diffuse tubular structures. (**G**). Large arrows indicate chloroplast “pockets”. (**H**). Small arrows indicate vesicles that may contain HGSV2 particles.

To deepen the characterization of zonate chlorosis in Brazil, transmission assays were conducted with *Brevipalpus* mites. In Experiment I, mites collected directly from symptomatic *C. reticulata* fruits (Itj_01), later identified as *B. papayensis*, were transferred to fourteen arabidopsis leaves of four plants. Twelve days after infestation, 50% (7 out of 14) of the leaves tested positive for HGSV2 via RT-PCR (Figure 5A).

Additional experiments used two laboratory-established populations of non-viruliferous mites, *B. papayensis* and *B. yothersi*. They were allowed to acquire the virus from symptomatic *C. reticulata* leaves (CdA_15) and transferred to healthy arabidopsis plants. Across independent Experiments II and III, about 20% and 17% of the leaves tested positive, respectively (5 out of the 26 leaves, Experiment II; 11 out of the 66 leaves for Experiment III) (Figure 5A,B). The highest transmission rate was observed with mites taken directly from symptomatic citrus, which yielded seven positive samples out of fourteen (Experiment I).

Amplicons covering the full HGSV2 genome were obtained from one arabidopsis sample using primers based on the sequence of DMs_01, revealing >95% nucleotide identity with the reference isolate. Furthermore, TEM analysis of infected arabidopsis leaves revealed unique cytopathic effects not seen in healthy controls, including fibrillar material in the endoplasmic reticulum, diffuse tubular structures, and chloroplast “pockets” containing vesicles with potential HGSV2 particles (Figure 5C–H). These results confirm that *B. papayensis* and *B. yothersi* are competent vectors of HGSV2.

## 4. Discussion

Zonate chlorosis (ZC) was first described in Brazil in the 1930s [7]. In sweet oranges, it causes non-systemic chlorotic spots that can be mistaken for citrus leprosis (CL) [6,7,8]. Both CZ and CL were demonstrated to be transmitted by mites in the mid-20th century [10,11,37]. Currently, CL is widespread in Latin America and poses a major threat to citrus production, particularly in Brazil, Mexico and Colombia [5,38,39]. Consequently, research has largely focused on CL, while ZC has been neglected. In this study, we identified hibiscus green spot virus 2 (HGSV2, *Higrevirus waimanalo*) as the causal agent of ZC in Brazil, and report, for the first time, the presence of this virus outside of Hawaii, USA. Although identified in Brazil since the 1930s, ZC has few documented cases after the 1970s. However, after 2016, limited outbreaks were detected in at least three coastal regions of Brazil: in small citrus production areas in the State of Espírito Santo, and in the Citrus Germplasm Collections of Embrapa in the State of Bahia and of Epagri in the State of Santa Catarina. Low incidence of ZC may be partly due to underreporting, misidentification of symptoms or symptom masking by nutritional deficiencies or other diseases, such as CL.

Our combined HTS, Sanger sequencing, and RT-PCR on both contemporary (2016–2022) and historical herbarium samples from original ZC outbreaks (1937–1962) provided conclusive evidence for the causal agent of the disease. Detection in historical specimens, including one from a 1960s transmission experiment with *B. phoenicis* s.l., directly linked HGSV2 to classic ZC symptomatology and confirmed early biological evidence of mite transmission [11]. HGSV2 full genomes were recovered from DMs_01 (OR161047, OR161046, OR161045) and DMs_02, and partial genome of isolates Itn_02, CdA_01 and Cmp_01 isolates (Supplementary Table S2, accession numbers PX437934-PX437948). The genomic organization of the Brazilian isolates is largely conserved to that of isolate WAI_1-1, collected in 2012 [12]. The main difference between them is the number and size of the ORFs in the RNA3. In this regard, Brazilian HGSV2 isolates display the RNA3 organization similar to the Ohau and Maui HGSV2 isolates, detected in Hawaii in 2024 [15].

The strategy of sequencing the sRNAs fraction to investigate the virome of herbarium specimens has been previously applied to citrus leprosis samples [18,40]. sRNAs, the target molecules for sequencing, are produced by the natural gene silencing mechanism involved in viral defense and gene expression regulation. Due to their size, sRNAs are more stable over time than longer mRNA molecules. In this study, we successfully applied the same approach to investigate the etiological agent of ZC in herbarium specimens. HGSV2 detected in Cmp_01 showed >94% nucleotide identity with DMs_01 (Table 1). The results demonstrate that this higrevirus has been present in citrus samples in Brazil for near 100 years and confirm HGSV2 as the causal agent of ZC.

So far, HGSV2 has been reported only in three islands of Hawaii [12,15] and, with this work, in coastal regions of Brazil. In Brazil, the disease caused by HGSV2 was initially described in the States of Rio de Janeiro and São Paulo (municipalities of Taubaté, ~70 km from the sea, and Ubatuba, a coastal city). During the 1960s, ZC was also reported in citrus orchards of Cruz das Almas, Bahia (~80 km from the sea) [1,2,3,6,7,10]. In this study, we fully or partially sequenced isolates from Ubatuba, SP (Ubt_01, 1933) and Itanhaém, SP (Itn_02, 1937), confirming the presence of HGSV2 in coastal samples. Together, the Brazilian and Hawaiian reports suggest that ZC is strongly associated with sea-level environments, possibly reflecting environmental factors that shape virus-vector-plant interactions.

Previous studies reported *Brevipalpus* mites on *C. volkameriana* plants infected with HGSV2 (WAI_1-1) [12], supporting early evidence that *B. phoenicis* s.l. vectors ZC [11]. Subsequent taxonomic revisions have since recognized *B. papayensis* and *B. yothersi* as separate species in the *B. phoenicis* complex [34]. Here, we demonstrated that both species can transmit HGSV2 to arabidopsis plants. We also validated the classic transmission experiments of 1965 [11] by detecting HGSV2 in a herbarium sample (SPa_01) that had been subjected to experimental mite transmission. The vector capability of these species is not limited to higreviruses; *B. azores* is also a known vector of citrus bright spot virus (CiBSV, *Dichorhavirus australis*), a dichorhavirus that causes citrus leprosis [36], whereas *B. yothersi* and *B. papayensis* are vectors of several other *Brevipalpus*-transmitted viruses (BTVs) [41]. Taken together, our results, along with reports of *B. yothersi* and *B. papayensis* on HGSV2-infected hibiscus (*Hibiscus arnottianus*) shrubs in Hawaii, *B. papayensis* on infected *C. volkameriana* [12,42], and the transmission of HGSV2 by *B. azores* [15], provide critical evidence that confirm the role of different species of *Brevipalpus* mites as vectors of this higrevirus.

The uneven distribution of the *Brevipalpus* mite species may explain, at least in part, the epidemiology of ZC in Brazil. For example, *B. papayensis* is a rare species in commercial Brazilian citrus orchards, having been found only in non-commercial citrus areas [42,43]. In contrast, *B. yothersi* is widespread in citrus-growing regions of Brazil. The geographical restriction of a disease due to vector absence is common, as seen for the dichorhaviruses CiLV-N (*Dichorhavirus leprosis*) and CiCSV (*Dichorhavirus citri*), transmitted by *B. phoenicis* and *B.* aff. *yothersi*, respectively [17,44]. Finally, transmission efficiency may vary between populations of the same species, as reported for the interaction between CiLV-C and *B. yothersi* [45].

Altogether, our work resolves the longstanding question of the zonate chlorosis etiology in Brazil by identifying HGSV2 and confirming its transmission by Brevipalpus papayensis and B. yothersi. The natural host range of HGSV2 in Brazil is broad, including multiple citrus species (*C. reticulata*, *C. clementina*, *C. deliciosa*, *C. aurantium*, *C. sinensis*, *C. limon*, *C. limonia*, *C. aurantiifolia* and *C. maxima*), as well as hybrids like Piedmont Tangelo (*C. sinensis* × *C. paradisi*) [10,11]. Given this wide host range, an eventual change in vector population or agricultural practices could potentially alter its epidemiology, posing potential threats to the Brazilian citriculture. Finally, our results also demonstrate that viruses from all three genera of the family *Kitaviridae*, including cileviruses [13] and blunerviruses [46], occur in Brazil.

## Figures and Tables

**Table 1:** Summary of the set of *Citrus* spp. samples analyzed in this study.

Year of Collection	Host Species	Isolate Identification	Place of Collection ^a^	Experimental Use
Fresh samples				
2016	*C. sinensis*	CdA_01	Cruz das Almas, BA	HTS (mRNA)
2016, 2016, 2021	*C. sinensis*	CdA_03, CdA_04, CdA_12	Cruz das Almas, BA	RT-PCR, mite collection and identification
2016	*C. deliciosa*	CdA_07	Cruz das Almas, BA	RT-PCR
2016, 2021	*C. reticulata*	CdA_08, CdA_15	Cruz das Almas, BA	RT-PCR, inoculum for mite transmission (Experiment II and III)
2016	*C. clementina*	CdA_09	Cruz das Almas, BA	RT-PCR
2016	*C. aurantium*	CdA_10	Cruz das Almas, BA	TEM, RT-PCR
2016	*Citrus* sp.	VNI_01	Venda Nova do Imigrante, ES	RT-PCR
2017, 2017	*C. reticulata*	DMs_01, DMs_02	Domingo Martins, ES	HTS (mRNA)
2017	*C. sinensis*	DMs_03	Domingos Martins, ES	RT-PCR, mite collection and identification
2021	*C. sinensis* × *C. paradisi*	CdA_14	Cruz das Almas, BA	RT-PCR
2021	*C. limon*	CdA_17	Cruz das Almas, BA	RT-PCR
2022	*C. reticulata*	Itj_01, Itj_02	Itajaí, SC	RT-PCR, mite identification and viral transmission (Experiment I)
Herbarium samples				
1933	*C. reticulata*	Ubt_01	Ubatuba, SP	HTS (small RNA)
1936	*Citrus* sp.	Cmp_01	Campinas, SP	HTS (small RNA)
1937	*Citrus* sp.	Itn_02	Itanháem, SP	HTS (small RNA)
1962	*C. limonia*	SPa_01	São Paulo, SP	HTS (small RNA)

^a^ Brazilian states abbreviation: Bahia (BA), Espírito Santo (ES), Santa Catarina (SC) and São Paulo (SP).

**Table 2:** Nucleotide sequence identities between the Brazilian isolate of HGSV2 DMs_01 and other isolates of the genus *Higrevirus*. The asterisk (*) marks isolates from which we could not recover full genome sequence.

Virus	GenBank Accession Number	Genomic Segments		
HGSV2 DMs_01	OR161045, OR161046 and OR161047	RNA1 (%)	RNA2 (%)	RNA3 (%)
HGSV2 DMs_02	PX437934, PX437935 and PX437936	100	99.9	99.9
HSGV2 CdA_01	PX437943, PX437944 and PX437945	94.9 *	87.5 *	92.1 *
HGSV2 Itn_02	PX437940, PX437941 and PX437942	87.6 *	93.8	90.3 *
HGSV2 Cmp_01	PX437946, PX437947 and PX437948	91.1 *	95.7 *	96.2 *
HGSV2 WAI_1-1	NC016141, NC016143 and NC016142	97.6	93.3	92.5
HGSV2 UHM2019	OQ689785, OQ689786 and OQ689787	97.7	93.4	93.1
HGSV2 Maui_2021	OQ689788, OQ689789 and OQ689790	97.6	92.6	93.1
PixVX	MT334620, MT334619 and MT334618	54.2	51.5	47.6
PaHLV	OP324809, OP324810 and OP324811	53.8	48.7	43.2

## Data Availability

The genomic sequences of viruses described in this study are available at the GenBank database under accession numbers PX437934-PX437948.

## Supplementary Materials

The following supporting information can be downloaded at: https://www.mdpi.com/article/10.3390/v17111428/s1, Table S1: Quality report of the raw sequencing data of all isolates of HGSV2 analyzed in this work through high throughput sequencing. The Q30 analysis focused on reads of 10–34 bp as those were the target molecule size for sequencing. Table S2: Quantity and size of contigs obtained in the high throughput sequencing (HTS) of all analyzed isolates of HGSV-2 in this work. Table S3: Primers developed for the validation of HTS-obtained sequences of Brazilian HGSV-2 isolates through RT-PCR and Sanger sequencing and for standard detection of HGSV-2.
