# Supplementary material for: Historical and Contemporary Evidence Confirms a Higrevirus as the Causal Agent of Citrus Zonate Chlorosis in Brazil
Source: Viruses. 2025 Oct 28;17(11):1428. doi: 10.3390/v17111428 (PMC12656880; doi:10.3390/v17111428)
Supplement: Supplementary file 1 [file viruses-17-01428-s001.zip › viruses-3934379-supplementary.pdf]

Supplementary Table S1: Quality report of the raw sequencing data of all isolates of HGSV2 analyzed in this work through high throughput sequencing. The Q30 analysis focused on reads of 10-34 bp as those were the target molecule size for sequencing.

Isolate identification	N° of reads per library	%GC	Sequence lengths	Q30 of reads of 10-34 bp
HGSV2_Ubt_01	9.477.677	58	8-142	28.22
HGSV2_Spa_01	7.417.994	57	8-138	28.21
HGSV2_Itn_02	11.598.792	58	8-140	28.21
HGSV2_Cmp_01	9.036.575	57	8-140	28.21
HGSV2_CdA_01	13.7741.658	44	35-126	36
HGSV2_DMs_01	15.196.586	44	35-126	36
HGSV2_DMs_02	14.553.789	44	35-126	36

Supplementary Table S2: Quantity and size of contigs obtained in the high throughput sequencing (HTS) of all analyzed isolates of HGSV-2 in this work.

Isolate identification	Contigs			Size of contigs and assembled genomic segments(nt)			Genome size and coverage (%)		
	RNA 1	RNA 2	RNA 3	RNA1	RNA2	RNA3	RNA 1	RNA 2	RNA3
HGSV2_DMs_01	1	1	1	8384 / 8384	3210 / 3210	3184 / 3184	100	100	100
HGSV2_DMs_02	1	1	1	8375 / 8375	3215 / 3215	3188 / 3188	99	98	99
HGSV2_CdA_01	5	2	1	228-807 / 8157	465-1118 / 3155	1086 / 2885	93	98	97
HGSV2_Ubt_01	1	3	0	260	369-1757	-	3	54	-
HGSV2_Spa_01	1	5	0	237	349-594	-	3	18	-
HGSV2_Itn_02	5	3	2	484-5050 / 7898	487-1428 / 3174	1290-1800 / 3188	94	99	100
HGSV2_Cmp_01	11	1	4	255-1266 / 8367	3228 / 3228	296-1090 / 3196	99	100	100

Supplementary Table S3: Primers developed for the validation of HTS-obtained sequences of Brazilian HGSV-2 isolates through RT-PCR and Sanger sequencing and for standard detection of HGSV-2.

Primer	Sequence (5′-3′)	Primer	Sequence (5′-3′)
RNA1			
1F	TCACAAGGCTTCTAACAAGC	1R	CGAAACATGTCCTCATTGTA
2F	GAACAACTATATTCGGTACCAC	2R	CAATCTATGTCTCCTTTCACC
3F	TGTTTGATCAAGTGATGAGG	3R	TGGAATGCTTCTTTTGATCT
4F	GGATTCGGTGTATGAGAAGA	4R	GGATAGAAAGAAGGAACACG
5F	ATGAGGATGATCCATTGGT	5R	GACCATCGTAGACCAAATCT
6F	GTCTGGTTTTTGCTCCTCTT	6R	GATCATCCTCAACAACTCC
7F	ATGCTTACGGGTTGTGAGATA	7R	GTGCAGCATCCAAAATCT
8F	GTTTGAGGACATATCGAAGG	8R	ACAAATCCTCCTTCACAAAA
9F	GCCTTTTAATAGGTCGTTCA	9R	GTCGTCACAAACAAACTTCA
10F	ACTGAGAACAAGGCGCAGAT	10R	TCAACAATGATTTCTTTATGC
11F	GTGCCGGATAGGGTAGTT	11R	CCTTCTCCAACACCAACA
RNA2			
1F	TCTTTTGGCTGGTCTAGAAG	1R	ATGAACAAACTGGGCCTAC
1Fa	GTTACTGTGACAGGTAACGT	1Ra	GAGCATGTTACTGACCAGAA
2F	GATATCCATATCGATCCTGTG	2R	CACAAACTTGGTCTTACCAC
3F	GATGAGGTTGATGACGATG	3R	GCAACCAGTTTCAAAGATG
3Fa	GTTTTGTGGGATGGTCCTT	3Ra	GTATATCAGCAGAGCGAACA
4F	GATATTACGCCCGGTAAGT	4R	AACAACAACCAACAAGATCA
5F	CGATATTTCAAGGTCAAGCG	5R	ATGGAGAGAAAGCACAAAA
		5Ra	AGAAGAAGACAGGAAACCAC
RNA3			
1F	AGGTTGTGTATTGGTTGTTG	1R	AGACAAGCTAGCCGAACTAC
1Fa	TTATCACCCTAGACGCTGTA	1Ra	ACGAAACACATTCACAATCG
1Fb	GCGAGATAGTGCTATCGC	2R	GCATAAACAAACCAGCAAT
1Fc	AATTCTGCTGCGGTGATACC	2Ra	AGCCAACAGATTCATCGG
2R	GGTTCTGATGTTGTCAGAGT	3R	GAGTAAACGTGCAAAGACAC
3F	TCTACGTAGGACGAGTGATG	4R	CGCGCTTTATAACTGGTAA
4F	GGTAGTTTACAATAAAGGTGAGG	4Ra	AGAAACATGCAAGAAACTCGG
4Rb	CTGCAAAACCCGCTTATTC	4Rc	CAGGGAAAAGAGAAAGGAAAA
